# Cooking fuels use and carotid intima-media thickness during early pregnancy of women in Myanmar

**DOI:** 10.1371/journal.pone.0236151

**Published:** 2020-07-29

**Authors:** Myo Min, Nutta Taneepanichskul

**Affiliations:** College of Public Health Sciences, Chulalongkorn University, Bangkok, Thailand; University of Perugia, ITALY

## Abstract

**Background:**

Fuels burned in households for cooking cause indoor air pollution, exposing those who are cooking. Despite the mounting evidence of the effects of fuels use on health, few studies focus on the effect of cooking fuels have on carotid intima-media thickness (CIMT), a surrogate atherosclerosis biomarker in the early stages of pregnancy. This study aimed to examine the association between the use of cooking fuels and CIMT during early trimester of pregnancy among cooking women in Myanmar.

**Methodology:**

In this cross-sectional study, a part of an ongoing birth cohort analysis, a total of 192 cooking pregnant women over 18 years with gestational weeks less than 18 were recruited from 15 rural health centers in Nay Pyi Taw from September to November 2019. Sociodemographic data, residential data, and fuels use data were collected with semi-structured questionnaires in face-to-face interviews. Anthropometric, hemodynamic, blood lipids, and ultrasound CIMT measurements were performed under standard protocols. Multiple linear regression was modeled to explore associations.

**Results:**

The study included 70 firewood fuel users, 26 charcoal fuel users, and 96 electricity fuel users. Following adjustments for potential confounding factors, firewood use was significantly associated with the increase of all CIMT analyzed. Importantly, a greater increase of mean CIMT of the right common carotid artery (RCCA; β = 0.033 mm; 95%CI: 0.006, 0.058; P<0.05) had significant association with charcoal use compared to firewood use (β = 0.029 mm; 95%CI: 0.010, 0.049; P<0.05).

**Conclusions:**

Our findings demonstrate that the indoor use of cooking fuels that cause indoor air pollution, such as firewood and charcoal, is a considerable risk factor for human health and is associated with increased CIMT, wherein charcoal use contributes to more increase of mean CIMT of the RCCA. Measures to prevent health risks related to the use of such fuels should be instituted early on during pregnancy.

## Introduction

Over 3 billion people in the world and more than 90% of people in developing countries including Myanmar mainly rely on biomass fuels for cooking, and the household combustion of these fuels in inefficient and ineffective cook stoves is a primary source of indoor air pollution (IAP) [1]. Burning solid fuels emits hazardous pollutants such as carbon monoxide (CO), carbon dioxide (CO_2_), particulate matters (PM_10_ and PM_2.5_), sulphur dioxide (SO_2_), benzene, formaldehyde, acrolein, and polycyclic aromatic hydrocarbons (PAHs) [2]. Harmful health effects from exposure to these pollutants and complexly formed compounds within smokes include high blood pressure [3, 4], respiratory illness [5, 6], nervous systems diseases [7] risks of preeclampsia [8, 9] and low birth weight [10, 11]. Annually, over 3.8 million premature deaths across the globe are due to cardiovascular diseases, chronic obstructive pulmonary disease, heart diseases, strokes and cancers, which are all attributable to indoor air pollution [12].

Many studies have revealed that air pollutants are involved in the arterial wall’s pathology by activating a cascade of inflammatory mediators in the body [13, 14]. Atherosclerotic changes can be detected early from the level of CIMT, which is the thickness feasibly measured by an ultrasound at the innermost layers of the carotid artery wall, i.e., between medial-adventitial and intimal-luminal interfaces [15]. CIMT value, an arthrosclerosis biomarker, is applicable to predicting cardiovascular vascular events and preeclampsia because vascular changes are recognizable as a window for the risks of developing cardiovascular disease (CVD) later in life [16]. However, increased CIMT has been found to be associated with many traditional risks factors such as age, sex, race, smoking, alcoholic beverages, exercise, blood pressure, abnormal lipid profiles, diet, consuming lipid lowering agents, glycemia, hyperuricemia, and obesity-related conditions. Novel risks factors are genetics, some inflammatory diseases, lipid metabolism, blood cells functions, job stress and vitamin-D [17]. Furthermore, nulliparity [18], bearing of more children [19], and diabetic condition [20] are found to be associated with CIMT. Based on a significant role of CIMT, there is a need of more research on CIMT during pregnancy so as to apply CIMT values for the benefits of better maternal health.

Throughout Myanmar, firewood (69.2%), charcoal (11.8%), and electricity (34.0%) are used in cooking [21]. Also, as Myanmar’s traditional primary cooks, women are often exposed to unhealthy smokes from fuels combustion. To date, literature on the association of fuels use with CIMT levels, particularly in the early stages of pregnancy, is limitedly available. Hence, the purpose of our study was to investigate the association between the use of different types of cooking fuels and maternal carotid intima-media thickness in early pregnancy among cooking women in Nay Pyi Taw area of Myanmar.

## Materials and methods

### Study design, population and ethical approval

This cross-sectional study, a part of an ongoing birth cohort to examine associations between the use of cooking fuels and CIMT and preeclampsia among pregnant women who were the primary cooks in their households, was conducted in Nay Pyi Taw (NPT) Area where firewood (53.8%), charcoal (11.1%) and electricity (34%) are used as the main energy sources for cooking [22]. Data were collected between September 2019 and November 2019. Nay Pyi Taw is the new capital city of Myanmar established over 10 years ago; the total population is 1,160,242 with 32% urban dwellers. The area of 27,248 square miles (70,572 square kilometers) was composed of eight townships in two districts. In this area, women of reproductive age, 15–49 years, totaled 307,391, and the total number of deliveries each year accounted for 6,261 in 2014, 7,078 in 2015 and 9,287 in 2016 [23]. Rural health centers (RHC) are basic health units where pregnant women register their pregnancies early and receive maternal, antenatal, and immunization services. The following 15 RHCs were selected depending on the availability of eligible participants and favorable distances: 1) Pyi san aung, 2) Nyaung bingyisu, 3) Aung zabu, 4) Thaedaw, 5) Tabyae gone, 6) Koeywar tabyae gone, 7) Ayemyint tharyar, 8) Ottrathiri, 8) Mingone, 10) Kwum chansu, 11) Ywar thit, 12) Medee, 13) Alar, 14) Tayet kone, and 15) Thit poke pin. The purposive sampling was used to recruit a total sample of 192 pregnant women from these RHCs during September to November, 2019. The inclusion criteria for pregnant women were as follows: 1) <18 weeks gestation; 2) age >18–45 years; 3) the primary cooks in the household, cooking at least once a week; 4) the use of any type of fuels (firewood, coal, charcoal, gas, LPG, electricity) in cook stoves for cooking purposes for at least six months; and 5) currently living in a household in NPT and giving confirmation to continue living in NPT until delivery. Exclusion criteria were 1) hypertension (≥140/90 mmHg); 2) proteinuria ≥1+; 3) urinary tract infections and 4) known renal and liver diseases. The sample size was estimated by using eipinfo software version 7.2.2.16, based on preeclampsia expected as 8% among the clean fuels user [24] and odds ratio of preeclampsia in biomass user 3.87 [9] with ɑ = 0.05 and 80% power. Addition of 10% attrition rate to 174 has resulted in the total sample of 192. The study protocol was reviewed and approved by the Research Ethics Review Committee for Research Involving Human Research Participants, the Health Sciences Group, Chulalongkorn University (RECCU; COA No.113/2019), and the Institutional Review Board, University of Public Health, Yangon UPH-IRB (2019/Research/34). The written informed consent of participants was obtained by providing information about study objectives and the freedom to refuse or withdraw from the study anytime.

### The carotid intima-media thickness measurement

The CIMT was measured in centimeters of both the RCCA and the Left Common Carotid Artery (LCCA). Offline images captured with linear probes (7.5 MHz) of high-resolution B-mode portable MEDISON ultrasound machines (SAMSUNG MEDISON CO., LTD, Korea) were taken by only one experienced radiologist blinded to exposure status of the participants. Acknowledging possible changes of CIMT during cardiac cycle and substantial dependency on technician skills in acquiring images, the radiologist followed and maintained standard protocols at all times [15]. Procedurally, participants were properly positioned in a comfortable supine position with their heads rotated 45 degree toward the right, then left in alternative turns for clear visualization of the CIMT of both arteries [25]. The far wall thickness of both arteries was taken from anterior, lateral, posterior angles by ensuring that these images were visualized from a 10-millimeter distance proximal to the bulk of the common carotid arteries [15, 26]. The thickness of layers between medial-adventitial and intimal-luminal interfaces that is marked in double line density was the measurement of the CIMT values. Then, measured values of anterior, lateral, posterior sides of each common carotid artery (left and right) were averaged, and 1) combined mean CIMT (combined left and right mean CIMT), 2) mean CIMT of the LCCA and 3) mean CIMT of the RCCA were reported in millimeters.

### Questionnaires and inspection checklists for exposure assessment

Participants in face-to-face interview were questioned by four trained research assistants to collect required data. Questionnaires were validated for their content by three experts in the fields of obstetrics and gynecology, environmental health and public health. Semi-structured questionnaires in Myanmar (Burmese) included the sociodemographic characteristics of age (years), parity, gestational weeks, education level (≤primary, >primary), average monthly income (kyats); the residential characteristics of daily incense sticks burning (yes/no), daily mosquito coils burning (yes/no), and the presence of smokers at home (yes/no). Moreover, for exposure information, the fuels use characteristics of the frequency of cooking per week (number of times), duration of cooking per time (minutes), total cooking duration (years), current main fuels use and its use duration, and current cook stoves types in use and its use duration were collected. Additionally, information regarding kitchen (attached/separated home), cook stoves in use (charcoal/multipurpose stove/three stone open fire stove/ gas stove/ electric stove), and chimney (presence/absence) [27] were collected in an inspection checklist by research assistants.

### Clinical assessments

All clinical assessments were performed at RHCs on the appointed dates and times. Anthropometric measurements focused on body weight, which was measured with a TANITA digital weighing scale. The participants wore light clothing but no footwear. Height was measured with a measuring tape designed for height measure. Body Mass Index (BMI) was then calculated by weight in kilogram divided by height in meter squared.

Hemodynamic measurements were blood pressure (systolic and diastolic) and heart rates, which were measured with OMRON automatic blood pressure monitors (HEM-7130). Appropriate cuff size was used on the right arm at a sitting position to measure thrice following complete rest. After three measurements, an average blood pressure was then reported in millimeters of mercury (mmHg).

Laboratory measurements included random blood sugar (mg/dl) and lipids analysis (mg/dl) in a non-fasting state [28]. Participants were well-informed about the purpose and procedures of samples collections; then, 5 milliliters of venous blood and adequate amount of urine samples were collected at the RHC by following universal precautions during sample collections. All collected samples were kept in separate cool boxes and sent on the same day to Department of laboratory at Zabuthiri Specialist Hospital where all samples were analyzed under standard laboratory procedures.

### Statistical analysis

All data were analyzed with SPSS software for Windows (IBM SPSS, version 22, Armonk, NY, USA). Continuous variables were presented in mean and standard deviation. Categorical variables were reported in number and percentage. To examine differences of the general characteristics between fuels use groups (firewood, charcoal, electricity), the one-way analysis of variance (ANOVA) test was used for continuous data, and either chi-square test or Fisher’s exact test was applied as was appropriate for categorical data. Multivariable linear regression models were used for examining associations between the use of types of cooking fuels and CIMT (combined mean CIMT, mean CIMT of the LCCA, mean CIMT of the RCCA). Model^a^ was adjusted for covariates of pregnancy-related factors (parity, gestational weeks, BMI), and Model^b^ was adjusted for factors associated with CIMT (age, diastolic blood pressure, heart rate, daily incense sticks burning (yes/no), random blood sugar, low density lipoprotein cholesterol). Factors adjusted for Model^a^ and Model^b^ were in the fully adjusted model. Potential covariates were selected on the basis of evidences in recent publications [29–31].

## Results

This study included a total of 192 pregnant women comprising 70 firewood users (36.46%), 26 charcoal users (13.54%) and 96 electricity users (50%). Basic characteristics are presented in Table 1. The mean age in years (SD) of pregnant women were 26.94 (6.35) overall, 27.57 (6.37) in the firewood group, 27.92 (7.80) in the charcoal group, and 26.22 (5.87) in the electricity group. First time pregnancy, gestational weeks, education, income (kyats), cooking frequency per week, and heart rate differed significantly among fuels use groups (P<0.05). Other variables in sociodemographic, fuels use, residential characteristics, and clinical assessments were not significantly different. All participants responded they are not smokers. A chimney was absent in the kitchens of all participants’ households. There was no woman who was using coal, gas, LPG fuels in the current study population.

**Table 1:** Basic characteristics of pregnant women by cooking fuels use (n = 192).

Socio-demographic characteristics	Total (n = 192)	Solid fuel user (n = 96)		Non-solid fuel user (n = 96)	P-value
		Firewood (n = 70)	Charcoal (n = 26)	Electricity (n = 96)	
Age years, mean (SD)	26.94 (6.35)	27.57 (6.37)	27.92 (7.80)	26.22 (5.87)	0.280^a^
Parity, n (%)					0.019^b^
Parity = 0	117 (60.9)	36 (51.4)	13 (50.0)	68 (70.8)	
Parity ≥ 1	75 (39.1)	34 (48.6)	13 (50.0)	28 (29.2)	
Gestational weeks by USG, mean (SD)	14.38 (3.32)	15.26 (2.58)	14.42 (3.42)	13.72 (3.62)	0.012^a^
Education level, n (%)					<0.001^b^
> Primary	104 (54.2)	25 (35.7)	15 (57.7)	64 (66.7)	
≤ Primary	88 (45.8)	45 (64.3)	11 (42.3)	32 (33.3)	
Occupation, n (%)					0.798^b^
House-wife	107 (55.7)	39 (55.7)	16 (61.5)	52 (54.2)	
Others	85 (44.3)	31 (44.3)	10 (38.5)	44 (45.8)	
Monthly family income (kyats), n (%)					0.021^b^
> 200,001	91 (47.4)	24 (34.3)	15 (57.7)	52 (54.2)	
≤ 200,000	101 (52.6)	46 (65.7)	11 (42.3)	44 (45.8)	
**Residential characteristics**					
Kitchen Type (n, %)					0.115^c^
Home attached	148 (77.1)	54 (77.1)	24 (92.3)	70 (72.9)	
Separated	44 (22.9)	16 (22.9)	2 (7.7)	26 (27.1)	
Daily incense sticks burning, n (%)					0.061^b^
Yes	140 (72.9)	58 (82.9)	18 (69.2)	64 (66.7)	
No	52 (27.1)	12 (17.1)	8 (30.8)	32 (33.3)	
Daily mosquito coils burning, n (%)					0.142^b^
Yes	42 (21.9)	10 (14.3)	6 (23.1)	26 (27.1)	
No	150 (78.1)	60 (85.7)	20 (76.9)	70 (72.9)	
Smokers at home, n (%)					0.833^b^
Yes	88 (45.8)	31 (44.3)	11 (42.3)	46 (47.9)	
No	104 (54.2)	39 (55.7)	15 (57.7)	50 (52.1)	
**Fuels use**					
Cooking frequency at household per week, mean (SD)	11.14 (4.68)	11.97 (4.20)	12.04 (3.45)	10.29 (5.16)	0.041^a^
Cooking duration per time (min), mean (SD)	67.63 (38.58)	71.93 (36.94)	62.31 (35.17)	65.94 (40.65)	0.463^a^
Fuels use duration years, mean (SD)	10.87 (5.73)	11.71 (7.04)	9.35 (7.44)	10.67 (3.78)	0.178^a^
**Clinical assessments**					
Body Mass Index (kg/m^2^), mean (SD)	22.62 (4.81)	22.61 (4.24)	24.22 (7.29)	22.18 (4.32)	0.163^a^
Heart rate per minute, mean (SD)	90.73 (11.91)	88.67(10.85)	96.31 (12.33)	90.72 (12.16)	0.019^a^
BP (mmHg), mean (SD)					
Systolic Blood Pressure	105.55 (11.73)	103.91 (11.35)	103.62 (16.07)	107.26 (10.45)	0.129^a^
Diastolic Blood Pressure	67.82 (7.99)	66.49 (7.61)	70.19 (9.47)	68.16 (7.74)	0.110^a^
Random blood sugar (mg/dl), mean (SD)	89.09 (17.09)	88.53 (17.66)	89.94 (15.22)	89.28 (17.29)	0.928^a^
Random blood cholesterol (mg/dl), mean (SD)					
Total cholesterol (up to 220)	190.95 (33.64)	187.79 (29.89)	191.82 (33.09)	193.02 (36.42)	0.610^a^
HDL cholesterol (35–45)	55.39 (14.69)	57.29 (15.39)	50.81 (11.86)	55.24 (14.72)	0.156^a^
LDL cholesterol (<150)	108.42 (27.36)	103.31 (24.47)	111.16 (28.12)	111.41 (28.84)	0.146^a^
Triglycerides cholesterol (< 200)	136.24 (66.87)	133.77 (66.94)	151.24 (80.75)	133.98 (62.84)	0.472^a^
Cholesterol and HDL ratio	3.91 (2.67)	4.15 (4.17)	3.95 (1.12)	3.73 (1.13)	0.602^a^

^a^ = One-way analysis of variance.

^b^ = Chi-square test.

^c^ = Fisher’s exact test.

Comparing mean (SD) of the respective CIMT, combined mean CIMT 0.424 (0.051) mm was the highest in firewood users, and 0.421 (0.043) mm in charcoal users was higher than electricity users’ CIMT 0.394 (0.04) mm. Statistically significant differences in the mean values among three groups were observed (p<0.001). For mean CIMT of the LCCA, the thickness in firewood users 0.415 (0.048) mm was higher than those of charcoal users 0.408 (0.042) mm and electricity users 0.389 (0.046) mm, which were significantly different (P = 0.001). A similar finding was found in mean CIMT of the RCCA where CIMT levels in firewood users 0.432 mm (0.064) and in charcoal users 0.435 (0.054) mm were greater than level in electricity users 0.400 (0.057) mm. Significant differences were achieved (P = 0.001) among three fuels user groups, as seen in Table 2.

**Table 2:** CIMT of common carotid arteries by fuels use.

CIMT (mm), mean (SD)	Total (n = 192)	Solid Fuels (n = 96)		Non-solid fuels (96)	P-value
		Firewood (n = 70)	Charcoal (n = 26)	Electricity (n = 96)	
Combined mean CIMT	0.409 (0.049)	0.424 (0.051)	0.421 (0.043)	0.394 (0.046)	< 0.001^a^
Mean CIMT of the LCCA	0.400 (0.048)	0.415 (0.048)	0.408 (0.042)	0.389 (0.046)	0.001^a^
Mean CIMT of the RCCA	0.416 (0.061)	0.432 (0.064)	0.435 (0.054)	0.400 (0.057)	0.001^a^

^a^ = One-way analysis of variance.

**Fig 1:**
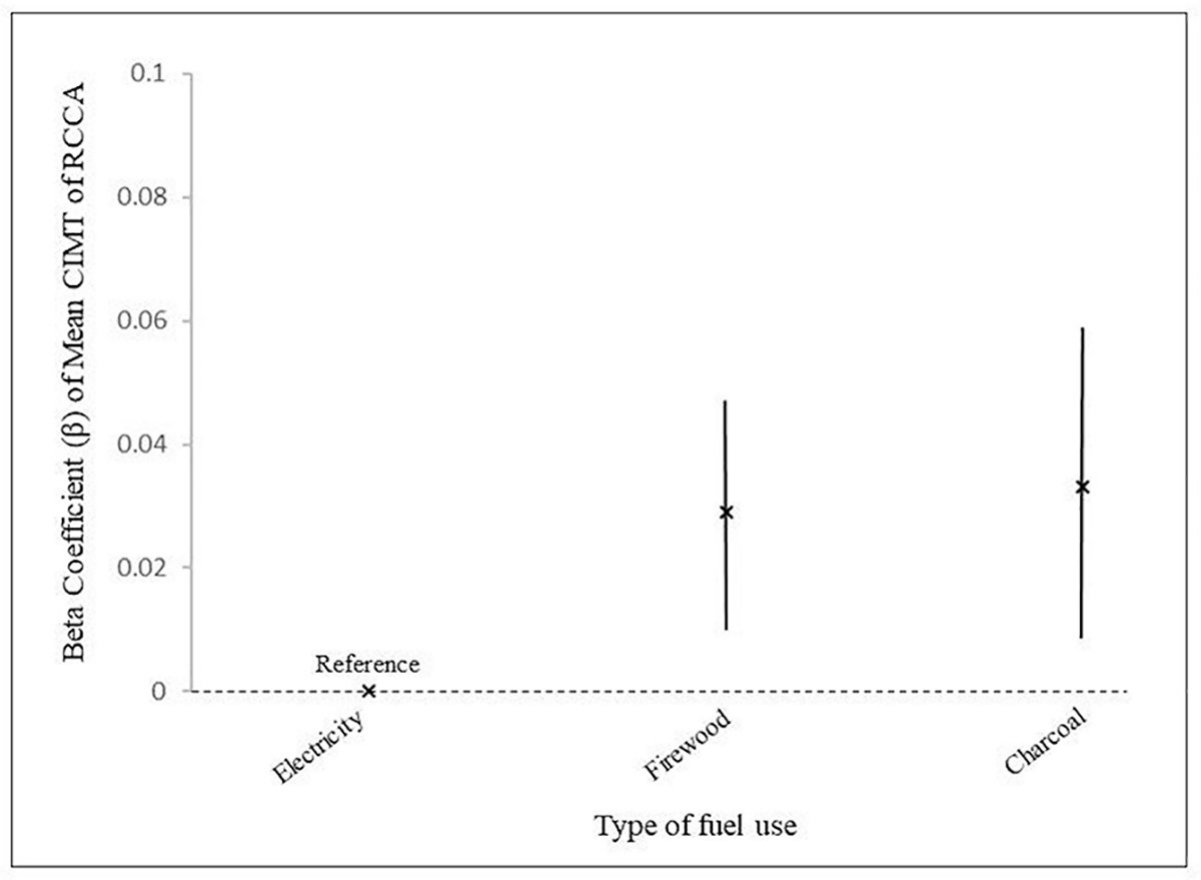
Beta coefficient of mean CIMT of the RCCA by type of fuels use.

Table 3 shows the association between fuels use type and the respective CIMT. Firewood use had significant associations with the increase of all CIMT analyzed. After adjusting for pregnancy-related factors and factors related to CIMT, mean CIMT of the RCCA consistently showed a significant association with firewood and charcoal use in all models. More specifically, charcoal use was independently associated with 0.033 mm increase in mean CIMT of the RCCA while firewood use was associated with 0.026 mm increase in mean CIMT of the RCCA, which were statistically significant (P<0.05). The coefficient of mean CIMT was shown in Fig 1.

**Table 3:** Multivariate linear regression model for combined mean CIMT, mean CIMT of the LCCA, and mean CIMT of the RCCA.

CIMT (mm)	Electricity		Firewood		P-value	Charcoal		P-value
	Coefficient	95% CI	Coefficient	95% CI		Coefficient	95% CI	
**Unadjusted model**								
Combined mean CIMT	Ref. category		0.029	0.014,0.044	**< 0.001**	0.027	0.006, 0.048	**0.012**
Mean CIMT of the LCCA	Ref. category		0.027	0.012,0.041	**< 0.001**	0.019	- 0.001,0.039	0.065
Mean CIMT of the RCCA	Ref. category		0.032	0.013, 0.050	**0.001**	0.035	0.009, 0.061	**0.009**
**Adjusted model** ^a^								
Combined mean CIMT	Ref. category		0.025	0.010, 0.040	**0.002**	0.021	0.001, 0.042	**0.044**
Mean CIMT of the LCCA	Ref. category		0.020	0.005, 0.053	**0.007**	0.013	- 0.007, 0.033	0.212
Mean CIMT of the RCCA	Ref. category		0.029	0.010, 0.048	**0.003**	0.030	0.004, 0.056	**0.025**
**Adjusted model** ^b^								
Combined mean CIMT	Ref. category		0.027	0.012, 0.042	**< 0.001**	0.025	0.004, 0.045	**< 0.001**
Mean CIMT of the LCCA	Ref. category		0.024	0.010, 0.039	**0.001**	0.017	- 0.003, 0.037	0.102
Mean CIMT of the RCCA	Ref. category		0.029	0.010, 0.048	**0.002**	0.033	0.007, 0.059	**0.012**
**Fully adjusted model** ^c^								
Combined mean CIMT	Ref. category		0.025	0.010,0.041	**0.001**	0.023	0.002, 0.044	**0.029**
Mean CIMT of the LCCA	Ref. category		0.022	0.006, 0.036	**0.006**	0.014	- 0.006, 0.034	0.167
Mean CIMT of the RCCA	Ref. category		0.029	0.010, 0.049	**0.003**	0.033	0.006, 0.058	**0.016**

^a^ = Adjusted for pregnancy related factors: parity, gestational weeks, BMI.

^b^ = Adjusted for factors associated with CIMT: age, diastolic blood pressure, heart rate, daily incense sticks burning (yes/no), random blood glucose, low density lipoprotein cholesterol.

^c^ = Adjusted for factors in Model^a^ and Model^b^.

## Discussion

Carotid intima media-thickness in early pregnancy is higher in firewood and charcoal use groups than in the electricity use group in this study population. Firewood use is associated with an increase of all CIMT analyzed. More importantly, our study demonstrated that a greater increase of mean CIMT of the RCCA has significant association with charcoal use compared to firewood use among cooking pregnant women in this country. These findings are valuable in Myanmar where the majority of population rely on solid fuels for cooking.

CIMT is increasingly measured not only because of the independent marker for cardiovascular diseases but also because of its usefulness for disorders in pregnancy and beyond [32]. A thickened carotid artery wall could be the result of structurally hypertrophic changes in response to metabolic insults from many factors, including pollutants exposed in a prolonged manner [13]. In our study, CIMT levels in the early stages of pregnancy in women who use different fuels in cooking were measured, and we found a significant association between cooking fuels used and increased CIMT. This finding is consistent with previous findings. Recent studies investigated the association of pollutants such as PM_2.5_ with CIMT, and they found that there was higher PM_2.5_ levels as well as a 0.04 mm higher CIMT in biomass fuels users as compared with non-biomass fuels users [4], which was reinforced by a meta-analysis in which it was concluded that each 5μg/m^3^ higher in PM_2.5_ exposure had a 1.04 μm per year greater CIMT progression [33]. Also, a study conducted in Peru showed that the biomass fuel user group had higher median PM_2.5_ concentration compared with clean fuel group (280 μg/m^3^ vs 14 μg/m^3^; p<0.001). Moreover, they elucidated that there was greater CIMT (mean difference = 0.03 mm) and a higher prevalence of carotid plague (OR = 2.6; 95%CI: 1.1, 6.0; p = 0.03) in biomass users compared to clean fuels users [34].

Our study also demonstrates that firewood use is associated with the increase of all CIMT analyzed and, a greater increase of mean CIMT of the RCCA has significant association with charcoal use compared to firewood use. Reasons could be firewood largely produces more particles among hazardous pollutants emitted from solid fuels burning. When particles are inhaled, they induce oxidation of surfactants in the lungs’ alveoli, which further activates the toll-like receptor 4. Again, that stimulates alveolar macrophages to produce pro-inflammatory cytokines. By passing alveolar epithelium, particles enter into circulation on a mechanism of transcytosis. Systemic inflammation then occurs by the interaction of particles with inflammatory mediators. Consequently, particles oxidize low density lipoprotein (LDLs) which is susceptibly up-taken by macrophages into the vessels’ wall resulting in plaque growth and thickened artery wall [14]. Charcoal, on the other hand, produces more carbon monoxide (CO). One study examined the effect of CO on blood vessels and found that CO lowers calcium concentrations in the smooth muscles of arteries that result in relaxation and the dilatation of blood vessels [35]. Hence, the CIMT level taken on the relatively relaxed artery could be higher than the level measured on the calcified (constricted) artery. It is worth nothing that calcification occurred more often on the right carotid artery while plaque formation and thickness were more prone on the left carotid artery, according to a study on 1,414 stroke-free patients aged ≥ 45 years [36]. However, as CO concentrations measurements, calcium biomarkers, calcification levels, and plaque were not assessed in this study, effect of CO on calcium levels of arteries that may result in the increase of CIMT of the RCCA warrants additional clarifications.

Furthermore, several recent studies in Mexico explored biomarkers related to biomass fuels use to investigate their implications and risks to human health. Among others, polycyclic aromatic hydrocarbons (PAHs), a chemical released from burning biomass fuels (woods) was investigated. Urinary 1-hydroxypyrene (1-OHP), an exposure biomarker for PAHs, was quantified and determined its associations with useful markers for cardiovascular diseases development. That 1-OHP has been found to be associated with serum adipocyte acid binding protein (FABP4), which is used in predicting metabolic diseases and CVDs [37]. Vascular dysfunction was also significantly associated with 1-OHP levels higher than 0.24 μmol/mol Cr, despite no association observed between 1-OHP and CIMT [38]. Another CVD risk marker, asymmetric dimethylarginine (ADMA), was also found to have significant associations with urinary 1-OHP level [39, 40] and paraoxonase 1 polymorphism (PON1 Q192R), a marker for genetic susceptibility [40]. Regarding further exploration on PAHs in women using different fuels, its metabolite 1-OHP in urine and plasma expression level of miR-126 and miR-155 were higher in wood users than LPG users, and it has also been indicated that plasma expression levels of miR-126 and miR-155 were significantly associated with urinary 1-OHP level [41]; these MicroRNAs play an important role in the process of modulating vascular malfunctions and arthrosclerosis [42].

The difference in carotid arteries’ wall thickness is also explained by different anatomical origins and pressures affected on respective arteries. Aortic arch pressure (hydrostatic pressure) exerts on the left artery as it originates from the aortic arch, and (dynamic pressure) from ascending aortic blood flow, it influences the right artery stemming out from innominate artery, which is an extension of the ascending aorta [43]. However, further confirmation is required for this possible relationship.

In our study, age has association with CIMT. Age is one of the important risk factors for not only thickened CIMT [31, 44] but also cardiovascular diseases such as strokes, myocardial infarctions, and ischemic heart diseases [45]. A prior study mentioned that normal median CIMT levels in women at age <30 years are 0.39 mm (25^th^ percentile), 0.40 mm (50^th^ percentile), and 0.43 mm (75^th^ percentile) for both right and left arteries [15]. In our study, the overall thickness of CIMT is 0.409 (0.049) mm for combined mean CIMT, 0.400 (0.048) mm for LCCA and 0.416 (0.061) mm for RCCA, which are comparatively in the normal ranges. However, setting and population differences to our study population should be taken into account, and the finding of increased CIMT in younger age (nearly 27 years) solid fuels users in our study underlines an important significance although CIMT level >0.90mm was considered to be high-risk for cardiovascular diseases [15].

Education level, monthly income, gestational weeks, and parity were not found to have significant associations with CIMT in our study. A Chinese study, on the other hand, found that higher education is associated with decreased CIMT [30]. Whatever the education level (>primary) among our participants, a true situation of unavailable electricity in some remote villages exists; therefore, they are to use only solid fuels without other choice. Also, women with lower income are more likely to use solid fuels rather than electricity simply because of inadequate funds for the costs of electricity and related appliances. All these conditions are met in our study, which may favor an increased risk of exposure to cooking smokes to possess increased CIMT. Regarding parity, in the findings of a study in Bangladesh, a number of children were positively associated with a CIMT increase of 4.5 μm per one birth (95%CI: 0.8, 8.1; p = 0.02) when analyzing 718 women (mean age 37.5 years) and 417 men (mean age 43.1 years). Again, their further analysis revealed that women with more children tend to have a higher thickness of CIMT, not men‒following adjustments for BMI, blood pressure, betel use or age [46]. Another study in Germany demonstrated U-shaped associations of nulliparity (adjusted mean CIMT = 0.81 mm (95%CI: 0.78, 0.84) and single parity (mean CIMT = 0.73 mm (95%CI: 0.72, 0.74) with increased CIMT when 1,195 women of 45 to 75 years of age were analyzed [18]. Since more pregnant women with zero parity are included in our study, it leaves cautious interpretation. With respect to gestational weeks with CIMT, according to a recent study in the US, CIMT is found to begin increasing from the second trimester throughout the course of pregnancy and postpartum [47]. In yet another study on pregnant women, CIMT in the first, second, and third trimesters were 0.47 (0.16) mm, 0.45 (0.14) mm, and 43.03 (0.12) mm in 56 women who later developed preeclampsia and 0.32 (0.09) mm, 0.33 (0.10) mm, 0.33 (0.09) mm in 618 women who developed non-preeclampsia [48]. Our study population with preeclampsia signs yet undetectable and 14.38 (3.32) weeks, one week after first trimester i.e., <13 weeks of gestation, have had thicker CIMT. Furthermore, blood pressure in pregnancy that remains lower until 18 weeks of pregnancy and a heart rate increase of 20% in early pregnancy [16] is in line with our study findings of low blood pressure and increased heart rates.

Our study results might be affected by some limitations. Small sample size recruited from the same area might limit the generalizability of results to other population due to their similar general characteristics. But, significant associations were investigated in the analysis. We did not measure specific pollutants to estimate their concentrations; however, cooking fuels use status of women in our study was confirmed by using inspection checklists by trained research assistants in addition to proper inclusion criteria for pollutants exposure. Additionally, previous studies worldwide to date rarely reported low emission of pollutants from solid fuels burnt inside households. Studies in Tanzania and Sri Lanka conducted indoor air pollutant measurements particularly at cook sites, and concentrations levels were CO geometric mean (SD) = 4.8 (46) ppm and PM_2.5_ = 40.5 (21.2) μg/m^3^ in Tanzania [49] and 48-hour CO concentrations (0.22 ppm to 8.66 ppm) and PM_2.5_ (33 μg/m^3^ to 940 μg/m^3^) in Sri Lanka [50]. These studies provided the real-time pollutant concentrations to which women can be exposed during cooking. Thus, one can extrapolate that pollution exposure is inevitable in this study population. Another limitation was we could measure only mean CIMT in this study although some scholars performed measuring mean and maximal CIMT [29, 34]. Moreover, CIMT measurement was done at a single time point, which requires follow-ups measurements to explore CIMT levels throughout pregnancy. Other than fuels use factors (cooking frequency, cooking duration, and fuels use years), we could not include information regarding other risks factors such as genetics, lifestyles, some biomarkers [17], and ambient pollutants [51] in our study, which might impact on the CIMT levels. Further studies should include these factors. The mean lipids levels were within normal limits, and the analysis was conducted on non-fasting blood, which is encouraged and widely accepted [28]. The CIMT was measured by only one radiologist, and gestational weeks confirmed by ultrasound strengthens validity of our findings.

## Conclusions

Among fuels used by pregnant women in Myanmar, our results demonstrated that firewood and charcoal fuels use were associated with increased CIMT of common carotid arteries. More specifically, charcoal use in comparison to firewood use revealed the association with more of an increase of CIMT of the RCCA. Therefore, using solid fuels, especially charcoal provided added evidence of risk factors for increased CIMT and the use of clean fuels and measures to mitigate risks of exposure to pollution in households specifically from solid fuels burning, is substantially recommended. Also, to objectively investigate exposure level and to estimate dose-response relationships, pollutants’ concentration measurements are required. Furthermore, a larger cohort with long-term follow-ups to examine the exposure effects of pollution on acute and later health problems of women burning solid fuels in cooking is advised.

## Supporting information

S1 File Questionnaires. (PDF)

S2 File Inspection checklists. (PDF)

S3 File Content validity. (PDF)

S4 File Data set. (XLSX)
