# Supplementary material for: Cooking fuels use and carotid intima-media thickness during early pregnancy of women in Myanmar
Source: PLoS One. 2020 Jul 29;15(7):e0236151. doi: 10.1371/journal.pone.0236151 (PMC7390349; doi:10.1371/journal.pone.0236151)
Supplement: S1 File — (PDF) [file pone.0236151.s001.pdf]

Appendix C

Questionnaire Form (Baseline)

Effect of cooking fuels use on increased maternal carotid intima media thickness and preeclampsia among self-cooking pregnant women in Nay Pyi Taw Area, Myanmar: A cohort study

Code No...…………..…………

Date……………………………

Part 1

A. Socio-demographic factors

(1) Completed age …………………………………… ……………………….. Years

(2) Gestation at entry …………………………………………………………….. Weeks

(3) Education level……………………………………………………………Grade

(4) Occupation

Government employee	Agriculturist	Merchant and trader	Company employee	Laborer	House-wife

(5) Monthly family income ………………………………………………………. Kyats

(6) Are you a current smoker?

No Yes, if yes, number of ………………………………… per day

(7) Do you drink alcohol?

No Yes, if yes, types …………………and ……… times per week

Part 2

B. Residential factors

(8) Dose your household burns incense sticks inside?

No Yes if yes, Non-daily Daily

(9) Does your household use mosquito coils inside?

No Yes if yes, Non-daily Daily

(10) Is there any family member who smokes at household?

No Yes

If yes, how many people smoking………, and total No.……………. Per day

Part 3

C. Cooking fuel use factors

(11) Self-cooking per week at household is……………………………………Times

(12) How long does cooking last per time when you cook? ………………….. Minutes

(13) How many years have you been doing self-cooking? …………………….Years

(14) What cooking fuels type do you **MAINLY** use in your household now? **Tick below**

Cooking fuels types		Main fuels in current use	Year of mentioned fuels used
Firewood	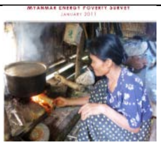 Picture source: Myanmar energy poverty survey, 2011		
Charcoal	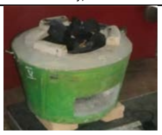 Picture source: Asia's Improved Cook Stoves		
Coal	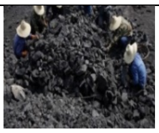 Picture source: http://www.investmyanmar.biz/infoNews.php?id=1398		
Gas	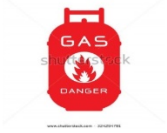 Picture source: Google		
LPG	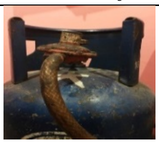		
Electricity	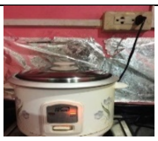		

(15) What cook stove (s) do you use at your household? **Tick below**

Stove type		Main stove in use	Years of cook stove used
Charcoal/ multipurpose stove	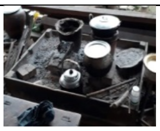		
Three-stones open fire stove	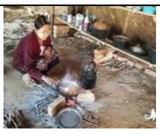 Picture source: Google		
Gas stove	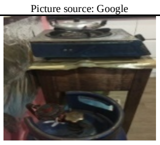		
Electric stove	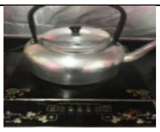		

...…………………………………**END, Thank you**…………………………………

နောက်ဆက်တွဲ (ဂ)

သုတေသနမေးခွန်းလွှာ (အချက်အလက်များကနဦးမေးမြန်းခြင်း)

မြန်မာနိုင်ငံ၊ နေပြည်တော်ဇရိယာအတွင်းရှိ ကိုယ်တိုင်ချက်ပြုတ်သည့်ကိုယ်ဝန်ဆောင် အမျိုးသမီးများတွင် အသုံးပြုသော လောင်စာအမျိုးအစား ပေါ်မူတည်၍ လည်ပင်းသွေးကြောမကြီးများထူထည်းမှုနှင့် ကိုယ်ဝန်ဆိပ်တက်ခြင်း ဆက်စပ်သက်ရောက်မှုကို လေ့လာသောသုတေသန

ကုတ်နံပါတ်…………………………………..

နေ့စွဲ…………………………………………..

အပိုင်း (၁)

(က) လူမှုစီးပွားဆိုင်ရာ အချက်အလက်များ

(၁) ယခုပြည့်ပြီးအသက်…………………………………………………………………………………. နှစ်

(၂) ယခုရောက်ရှိနေသောကိုယ်ဝန်ပတ်ပေါင်း………………………………………………………………. ပတ်

(၃) သင်ကြားခဲ့သောအမြင့်ဆုံးပညာအရည်အချင်း…………………………………………………………. တန်း

(၄) အလုပ်အကိုင်အမျိုးအစား

အစိုးရဝန်ထမ်း	စိုက်ပျိုးရေး	ကုန်သည်	ကုမ္ပဏီဝန်ထမ်း	အလုပ်ကြမ်းသမား	အိမ်ရှင်မ

(၅) မိသားစု တစ်လဝင်ငွေစုစုပေါင်း…………………………………………………………………………….. ကျပ်

(၆) သင်သည် လက်ရှိ ဆေးလိပ်သောက်သူ ဟုတ်ပါသလား။

မဟုတ်ပါ။ ဟုတ်ပါသည်။

ဟုတ်ပါကတစ်ရက်လျှင်သောက်သည့်ဆေးလိပ်စုစုပေါင်း…………………

(၇) သင်သည် အရက်သောက်သူ ဟုတ်ပါသလား။

မဟုတ်ပါ။ ဟုတ်ပါသည်။

ဟုတ်ပါက သောက်သည့်အရက် အမျိုးအစား………………………….။

တစ်ပတ်လျှင်သောက်သည့်ပမာဏစုစုပေါင်း………..………………… ခွက်

အပိုင်း (၂)

(ခ) နေအိမ်ဆိုင်ရာအကြောင်းအရာများ

(၈) သင်၏အိမ်အတွင်းတွင် အမွှေးတိုင်များထွန်းလေ့ရှိပါသလား။

မထွန်းပါ။ ထွန်းပါသည်။ ထွန်းပါက နေ့စဉ် နေ့စဉ်မထွန်းပါ။

(၉) သင်၏အိမ်အတွင်းတွင် ခြင်ဆေးခွေများ ထွန်းပါသလား။

မထွန်းပါ။ ထွန်းပါသည်။ ထွန်းပါက နေ့စဉ် နေ့စဉ်မထွန်းပါ။

(၁၀) သင့်မိသားစုဝင်များတွင် ဆေးလိပ်သောက်သူရှိပါသလား။

မရှိပါ။ ရှိပါသည်။ ရှိပါကဘယ်နှစ်ဦးသောက်ပါသလဲ……………………………။

တစ်ရက်လျှင်သောက်သည့် ဆေးလိပ်အရေအတွက်…………လိပ်။

အပိုင်း (၃)

(ဂ) ချက်ပြုတ်ရာတွင်သုံးစွဲသည့်လောင်စာအကြောင်းအရာများ

(၁၁) တစ်ပတ်လျှင် ဘယ်နှစ်ကြိမ်လောက် ကိုယ်တိုင်ချက်ပြုတ်လေ့ရှိပါသလဲ။ …………................................. ကြိမ်

(၁၂) ကိုယ်တိုင်ချက်ပြုတ်မှုတစ်ကြိမ်တွင် အချိန်မည်မျှကြာလေ့ရှိပါသလဲ။ ……………………………………… မိနစ်

(၁၃) ကိုယ်တိုင်ချက်ပြုတ်သည်မှာနှစ်ပေါင်း ဘယ်လောက်ကြာပြီလဲ။ …………………………………………….နှစ်

(၁၄) သင့်အိမ်တွင်ချက်ပြုတ်ရာ၌ **လက်ရှိတွင်** မည်သည့်လောင်စာအမျိုးအစားကို **အဓိက** အားဖြင့် သုံးပါသလဲ။ ဖော်ပြခဲ့သော ချက်ပြုတ်လောင်စာကို သုံးစွဲသည်မှာ **နှစ်ပေါင်း** ဘယ်လောက်ကြာပြီလဲ။

ချက်ပြုတ်လောင်စာအမျိုးအစားများ		လက်ရှိအဓိကသုံး လောင်စာ	အသုံးပြုသော နှစ်ပေါင်း
ထင်း	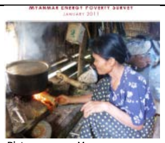 Picture source: Myanmar energy poverty survey, 2011		
မီးသွေး	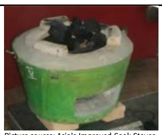 Picture source: Asia's Improved Cook Stoves		
ကျောက်မီးသွေး	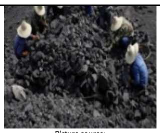 Picture source: http://www.investmyanmar.biz/infoNews.php?id=1398		
ဓါတ်ငွေ့	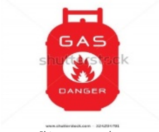 Picture source: google		
ရေနံဓါတ်ငွေ့ ရည်	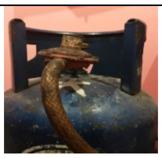		
လျှပ်စစ်	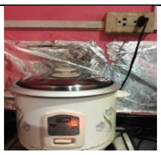		

(၁၅) သင့်အိမ် ၌ **လက်ရှိတွင်** မည်သည့်မီးဖိုကို **အဓိက** သုံးစွဲပါသလဲ။ ဖော်ပြခဲ့သောမီးဖိုကို သုံးစွဲသည်မှာ **နှစ်ပေါင်း**မည်မျှကြာပြီလဲ။

မီးဖိုအမျိုးအစားများ		လက်ရှိအဓိကသုံး မီးဖို	အသုံးသည့် ပြုနှစ်ပေါင်း
မီးသွေးနှင့် ဘက်စုံသုံးမီးဖို	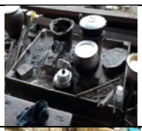		
သုံးချောင်းထော က်မီးဖို	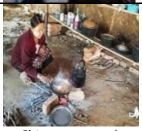 Picture source: google		
ဓါတ်ငွေ့ (သို့မဟုတ်) ဓါတ်ငွေ့ရည်မီးဖို	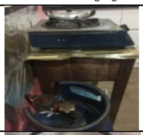		
လျှပ်စစ်မီးဖို	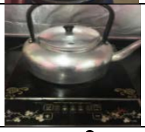		

……………………မေးခွန်းမေးမြန်းခြင်း ပြီးပါပြီ။ ကျေးဇူးအထူးတင်ပါသည်။…………………
