# Supplementary material for: Cooking fuels use and carotid intima-media thickness during early pregnancy of women in Myanmar
Source: PLoS One. 2020 Jul 29;15(7):e0236151. doi: 10.1371/journal.pone.0236151 (PMC7390349; doi:10.1371/journal.pone.0236151)
Supplement: S2 File — (PDF) [file pone.0236151.s002.pdf]

Appendix B

Inspection checklists

Effect of cooking fuels use on increased maternal carotid intima media thickness and preeclampsia among self-cooking pregnant women in Nay Pyi Taw Area, Myanmar: A cohort study

Code No……………

This form is to be used just for ascertainment of cooking fuels use at participant's households.

Cooking fuels use ascertainment checklists (baseline)			Remarks
Is cooking done at home?	Yes ✓	No	Examine cooking site, record
Self-cooking per week	Yes ✓	No	Ask it, record
What type of fuel do you MAINLY use for cooking? (tick only one below)			Ask and examine vicinity, record fuel types
Solid fuels group	Firewood	✓	
	Charcoal		
	Coal		
Non-solid fuels group	Gas		
	LPG		
	Electricity	✓	
Cook stove types in use	Charcoal/multipurpose stove	✓	Examine cooking site, record stoves types for evidence
	Three-stones open fire stove		
	Gas stove		
	Electric stove	✓	
Recent cooking activity	Presence	✓	
	Absence		
Kitchen	Attached home	✓	Examine cooking site, record it
	Not attached home		
Chimney	Presence	✓	
	Absence		

Definitions:

-Self-cooking refers to the practice of cooking done by the participant by using any one of fuels -Firewood, Coal, Charcoal, LPG, Electricity or Gas.

-Charcoal cook stoves - modeled stoves made of clay or metals

-Three stones cook stoves - cook stoves in three-stones and three-leg iron stoves

-Electric stoves – any stoves usable in connection to electric current

-Gas stoves – any stove used with LPG or gas fuel

✓ Indicator as seen in observation

Remarks-

Confirmed eligible **Solid** fuels user

Confirmed eligible **Non-Solid** fuels user

Name of inspector……………………………………………

Date……………………………………………………………...

သုတေသနတွင် ပါဝင်မည့်သူများအိမ်တွင်လောင်စာအသုံးပြုခြင်းကိုစီစစ်ရန် အသေးစိတ်အချက်အလက်များ

မြန်မာနိုင်ငံ၊ နေပြည်တော်ဧရိယာအတွင်းရှိ ကိုယ်တိုင်ချက်ပြုတ်သည့်ကိုယ်ဝန်ဆောင် အမျိုးသမီးများတွင် အသုံးပြုသော လောင်စာအမျိုး အစားပေါ်မူတည်၍ လည်ပင်းသွေးကြောမကြီးများထူထည်းမှုနှင့် ကိုယ်ဝန်ဆိပ်တက်ခြင်း ဆက်စပ်သက်ရောက်မှုကို လေ့လာသောသုတေသန

ပါဝင်သူကုတ်နံပါတ်...................

လောင်စာအသုံးပြုခြင်းကိုစီစစ်ရန် အသေးစိတ်အချက်အလက်များ (ကနဦးစစ်ဆေးခြင်း)			မှတ်ချက်
အိမ်တွင် ချက်ပြုတ်မှု	ရှိ ✓	မရှိ	မေးမြန်း မှတ်သားရန်
တပတ်လျှင် အိမ်တွင်ကိုယ်တိုင် ချက်ပြုတ်မှု	ရှိ ✓	မချက်ပါ	
အသုံးပြုသည့် **အဓိက** လောင်စာ (အမှန်ချစ်ရန်)			မေးမြန်း မှတ်သားရန်
လောင်စာခဲ	ထင်း	✓	ပတ်ဝန်းကျင်ကို ကြည့်ရှု၊ မေးမြန်း မှတ်သားရန်
	မီးသွေး		
	ကျောက်မီးသွေး		
လောင်စာခဲ မဟုတ်သောလောင်စာ	ဓါတ်ငွေ့		
	ရေနံဓါတ်ငွေ့ရည်		
	လျှပ်စစ်	✓	
အသုံးပြုနေသောမီးဖိုအမျိုးအစား	မီးသွေးနှင့်ဘက်စုံသုံးမီးဖို	✓	ပတ်ဝန်းကျင်ကို ကြည့်ရှု၊ မေးမြန်း မှတ်သားရန်
	သုံးချောင်းထောက်မီးဖို		
	ဓါတ်ငွေ့ (သို့မဟုတ်) ဓါတ်ငွေ့ရည်မီးဖို		
	လျှပ်စစ်မီးဖို	✓	
ချက်ပြုတ်မှုအထောက်အထားများ	တွေ့ရှိ	✓	
	မတွေ့ရှိ		
မီးဖိုချောင်တည်ရှိမှု	အိမ်နှင့်တွဲလျက်	✓	ပတ်ဝန်းကျင်ကို ကြည့်ရှု၊ မေးမြန်း မှတ်သားရန်
	အိမ်နှင့်မတွဲ		
မီးခိုးခေါင်းတိုင်	ပါရှိ	✓	
	မရှိ		

အဓိပ္ပါယ်သတ်မှတ်ချက်

–ကိုယ်တိုင် ချက်ပြုတ်သူဆို သည်မှာ ထင်း၊ မီးသွေး ၊ ကျောက်မီးသွေး၊ ဓါတ်ငွေ့၊ ရေနံဓါတ်ငွေ့ရည်၊ လျှပ်စစ်မီး စသည့်လောင်စာတစ်ခုခု ကို အသုံးပြုပြီး တပတ်လျှင် အနည်းဆုံး (၁) ကြိမ် အိမ်တွင် ကိုယ်တိုင် ချက်ပြုတ်သူ ချက်ပြုတ်ခြင်းကိုဆိုလိုခြင်း ဖြစ်သည်။

–မီးသွေးနှင့်ဘက်စုံသုံးမီးဖို – သံထည် (သို့မဟုတ်) ရွှံ့မြေဖြင့်ပြုလုပ်နိုင်သည်။

–သုံးချောင်းထောက်မီးဖို – သံထည် (သို့မဟုတ်) တခုခုဖြင့် သုံးချောင်းထောက်ပုံစံဖြစ်နိုင်သည်။

–ဓါတ်ငွေ့ (သို့မဟုတ်) ဓါတ်ငွေ့ရည်မီးဖို– ဓါတ်ငွေ့ (သို့မဟုတ်) ဓါတ်ငွေ့ရည်ဖြင့် သုံးသောမီးဖို။

–လျှပ်စစ်မီးဖို– လျှပ်စစ်မီးဖြင့် ချက်သောမီးဖို

– ✓ တွေ့ရှိမှုကိုမှတ်သားရန်

မှတ်ချက်

လောင်စာခဲအသုံးပြုသူ...

လောင်စာခဲမဟုတ်သောလောင်စာ အသုံးပြုသူ.................

ကြည့်ရှုစစ်ဆေးသူလက်မှတ်.......................................

ရက်စွဲ..
