# Supplementary material for: Cooking fuels use and carotid intima-media thickness during early pregnancy of women in Myanmar
Source: PLoS One. 2020 Jul 29;15(7):e0236151. doi: 10.1371/journal.pone.0236151 (PMC7390349; doi:10.1371/journal.pone.0236151)
Supplement: S3 File — (PDF) [file pone.0236151.s003.pdf]

CONTENT VALIDITY OF THE QUESTIONNAIRES

Title of the research: Effect of cooking fuels use on increased maternal carotid intima media thickness and preeclampsia among self-cooking pregnant women in Nay Pyi Taw Area, Myanmar: A cohort study

Researcher: Myo Min, MBBS, MMedSc (Disease Prevention & Control), MIPH

Doctorate student, Public Health Program, College of Public Health Sciences, Chulalongkorn University

Objective: To investigate associations between type of cooking fuels use and increased maternal carotid intima media thickness (CIMT) and preeclampsia among self-cooking pregnant women in Nay Pyi Taw area, Myanmar

Semi-structured interviewer administered questionnaire form is composed of (15) items for socio-demographic factors, residential factors and cooking fuels use factors. The Item-Objective Congruence Index (IOC) was analyzed for questionnaires' content validity testing by the following experts:

(1) Expert 1

Dr. Kyae Mhon Htwe (M.B.,B.S; M.Med.Sc (Biochemistry), MPH)

Deputy Director/Head, Quality Assurance Division, Department of Medical Research

Email: kyaehmon.htwe.dr@gmail.com

Ph: +9591-375457 ext: 501

(2) Expert 2

Dr. Tun Lin Maung (M.B.,B.S; M.Med.Sc (Obstetrics & Gynecology)

Specialist, Zabuthiri Specialist Hospital, Nay Pyi Taw

Email: drmgtunlinmg@gmail.com

Ph: + 9595121936

(3) Expert 3

Saowanee Norkaew, PhD

Faculty of Public Health, Thammasat University (Rungsit Campus)

The IOC index of the questionnaires was 0.92.

According to the three experts, the content validity in terms of IOC index 0.92 is approved, and the questionnaires are to be used for data collection for objectives mentioned above.
